# Ezh2 is involved in radial neuronal migration through regulating Reelin expression in cerebral cortex

**DOI:** 10.1038/srep15484

**Published:** 2015-10-26

**Authors:** Linnan Zhao, Jun Li, Yuanlin Ma, Jiutao Wang, Wen Pan, Kai Gao, Zhengrong Zhang, Tianlan Lu, Yanyan Ruan, Weihua Yue, Shanting Zhao, Lifang Wang, Dai Zhang

**Affiliations:** 1 Peking University Sixth Hospital/Institute of Mental Health, Beijing 100191, China; 2 National Clinical Research Center for Mental Disorders/Key Laboratory for Mental Health, Ministry of Health (Peking University), Beijing 100191, China; 3 College of Veterinary Medicine, Northwest A&F University, Yangling 712100, China; 4 National Laboratory of Biomacromolecules, Institute of Biophysics, Chinese Academy of Sciences, Beijing 100101, China; 5 Peking-Tsinghua Center for Life Sciences and PKU-IDG/McGovern Institute for Brain Research, Peking University, Beijing 100871, China; 6 Shenzhen Key Laboratory for Neuronal Structural Biology, Shenzhen Peking University-Hong Kong University of Science and Technology Medical Center, Shenzhen 518035, China

## Abstract

Radial migration of pyramidal neurons is an important event during the development of cerebral cortex. Neurons experience series of morphological and directional transitions to get to their final laminar positions. Here we report that the histone methyltransferase enhancer of zest homolog 2 (Ezh2) is involved in the regulation of cortical radial migration. We show that Ezh2 knockdown leads to disturbed neuronal orientation, which results in the impairment of radial migration. Further results reveal that this migration deficiency may be due to the derepression of *Reelin* transcription in the migrating neurons. Our study provides evidence that epigenetic regulation of Reelin by Ezh2 maintains appropriate Reelin expression pattern to fulfill proper orientation of migrating neurons.

The layered mammalian neocortex is mainly organized by groups of pyramidal neurons with different birthdates in the developing embryo^1^,^2^. Pyramidal neurons, generated from radial glia progenitor cells in the ventricular zone (VZ) and subventricular zone (SVZ), undergo series migration events to pass through the intermediate zone (IZ) and get to their final designations in the cortical plate (CP) in an “inside-out” manner^3^,^4^,^5^. The early-born neurons migrate through glia-independent somal translocation to form the pre-plate, while the later-born neurons migrate by glia-guided locomotion to pass through their predecessors then by leading process-relied terminal translocation beneath the marginal zone (MZ)^6^,^7^. The radial migration of these neurons is under subtle regulations of distinct genetic and environmental factors, and defects in this process lead to multiple brain structure abnormalities^8^,^9^, which may contribute to various neurological and mental diseases like periventricular heterotopia, fragile X syndrome, schizophrenia and bipolar disorder from both murine and human studies^10^,^11^,^12^,^13^,^14^.

The complexity of neuronal migration is under the prerequisite of dynamic changes in gene activation and repression, some of which relay on epigenetic regulations. Lines of evidences suggest that both DNA and histone modification mechanisms contribute to appropriate gene expression patterns that maintain proper migration events^15^,^16^,^17^,^18^. For instance, CoREST, a corepressor component of chromatin remodeling system that regulates neural gene expression during development, is responsible for the temporally appropriate onset of neuronal migration and morphological transition of neurons in this process dependent of the histone demethylase LSD1^19^.

Polycomb group proteins constitute another chromatin remodeling system that is indispensable for many cellular processes, such as proliferation, differentiation and maturation^20^,^21^. Ezh2, a member of polycomb repressive complex 2 (PRC2), is responsible for the tri-methylation of lysine residue 27 on histone H3 (H3K27me3) and maintains one important function of polycomb-mediated repression mechanism during development^22^. Missense mutation of *EZH2* may cause Weaver syndrome, characterized by accelerated growth and variable developmental delay^23^,^24^,^25^. Brain MRI study shows the existence of pachygyria in *EZH*-related Weaver syndrome patients, which is considered to be the consequence of abnormal neuronal migration^23^,^26^.

Functions of Ezh2 in cortical neural progenitor/stem cells have been investigated in recent years. Ezh2 deletion in cortical progenitor cells before onset of neurogenesis disturbs cortical development by marked changes in gene expression, thus alternating balance between self-renewal and differentiation^27^. In addition, Ezh2 is transcriptionally repressed by nuclear factor I/B (Nfib) and accountable for the deficit in differentiation of neural progenitor cell observed in Nfib-knockout mice^28^. Moreover, Ezh2 is also responsible for the neurogenic to astrogenic switching of neural progenitor cells^29^. However, whether and how Ezh2 is involved in the development of postmitotic cortical neuron is largely unknown. In this study, we show that Ezh2 is expressed by postmitotic migrating neurons, and by *in utero* manipulation of this gene we find that deficiency of Ezh2 in these cells lead to disturbed radial migration due to impairment in neuronal orientation, which may be caused by ectopic Reelin expression in these cells.

## Results

### Ezh2 is expressed in both proliferating and postmitotic migrating neurons

**Figure 1:**
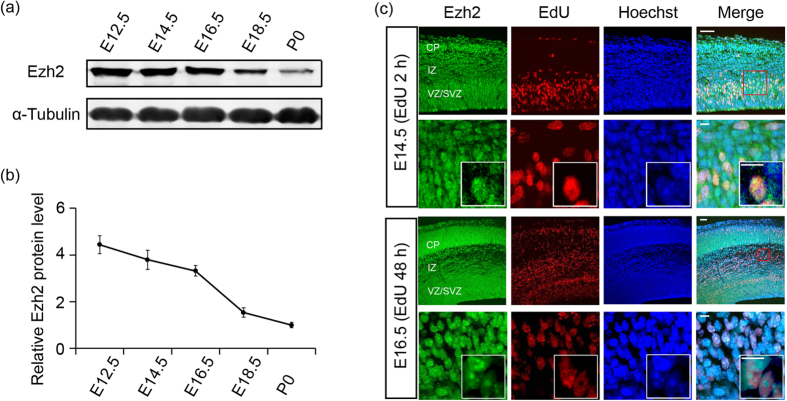
Ezh2 is expressed in both proliferating and postmitotic migrating neurons. (**a**) Immunoblotting for Ezh2 in mouse cortex lysates at the indicated ages shows that Ezh2 is expressed in both early and late stages of cortical development (cropped images, and full-length blots are presented in Supplementary Fig. S3a online). (**b**) Quantification of Ezh2 expression level done by normalization to that of α-Tubulin. *n *= 3 for each stage. Data represent mean ± SEM. (**c**) Two hours after EdU administration, EdU^+^ proliferating cells are strongly immunostained with Ezh2 antibody. Two days after the administration, Ezh2 is continuously expressed by EdU^+^ migrating cells. Scale bars: 50 μm and 10 μm (insets).

It has been reported that Ezh2 is expressed in cortical progenitor cells as early as embryonic day (E) 12, and the expression decreases over time until undetectable at postnatal day (P) 0^27^. To explore the role of Ezh2 in the cortical morphogenesis, we first examined its expression profiles in developing cortex in details. Western bolt analysis using a rabbit monoclonal antibody against Ezh2 showed that cortical lysates contained high level of Ezh2 at E12.5–E16.5 (Fig. 1a,b). At E12.5, the cortex is dominantly consisted of neural stem/progenitor cells and early-born neurons, while the proportion of progenitor cells decreases significantly during E14.5–E16.5, thus we speculated that Ezh2 may also be expressed by postmitotic neurons. The expression of Ezh2 decreased after E16.5 but was still detectable at P0 (Fig. 1a,b).

Progenitor cells at E14.5 generate pyramidal projection neurons in the VZ/SVZ, and these neurons undergo radial migration to get to their final designations in the upper layer of the cortical plate (CP). To determine whether Ezh2 is expressed in migrating neurons, immunofluorescence on brain sections from ethynyldeoxyuridine (EdU)-administrated embryos at E14.5 was performed. Two hours after the administration, EdU-labeled proliferating progenitor cells in VZ/SVZ were found to be intensely immunostained with Ezh2. Two days after the administration, most EdU-labeled cells were distributed in the IZ, and they were Ezh2-immunoactive also (Fig. 1c). In addition to the IZ, a few EdU/Ezh2-doubel labeled cells were also observed in the CP. These results not only confirmed the expression of Ezh2 in proliferating progenitor cells, but also showed its expression by postmitotic migration neurons.

### Loss of Ezh2 impairs cortical neuron migration

**Figure 2:**
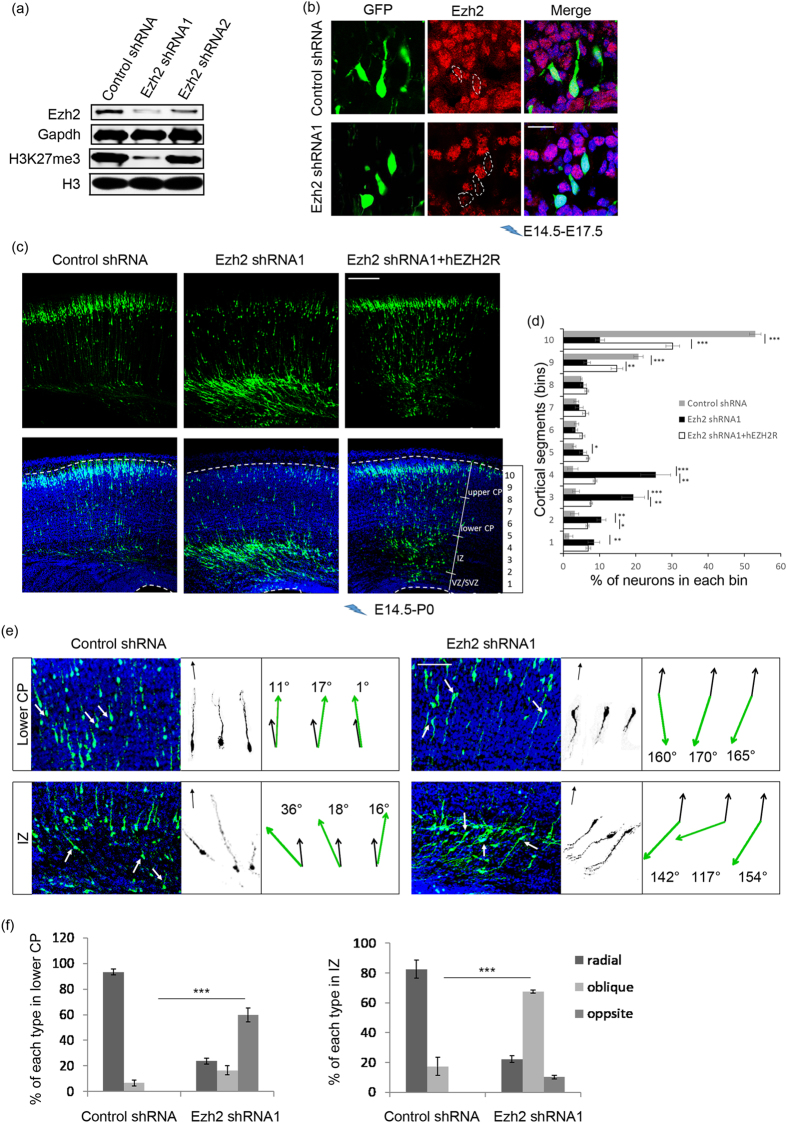
Knockdown of Ezh2 impairs neuronal migration. (**a**) Western blot of cultured E14.5 cortical neurons electroporated with plasmids for 3 days shows that relative to control shRNA, Ezh2 shRNA1 dramatically reduces the levels of both Ezh2 protein and H3K27me3, while Ezh2 shRNA2 shows less effect. (**b**) Ezh2 shRNA1 successfully reduces Ezh2 immunofluorescence *in vivo*. Dashed lines outline the nuclei of GFP-labeled neurons. Scale bar: 20 μm. (**c**) Representative images showing the E14.5 mouse cortices electroporated with indicated plasmids and examined at P0. Ezh2-knock down by shRNA1 leads to a reduction in the number of neurons in the CP, while an accumulation of neurons in the IZ. (**d**) Frequency distribution and quantification of GFP^+^ cells in ten equal bins (VZ 1 to CP 10). *n *= 6 for each group. Data represent mean ± SEM. One-way ANOVA plus Tukey’s multiple comparison tests; *p < 0.05; **p < 0.01; ***p < 0.001. Scale bar: 200 μm. (**e**) Left panel: images showing the distribution of electroporated neurons in lower CP and IZ of P0 mouse cortices. Middle panel: tracings of representative GFP^+^ neurons in each group. Black arrows indicate the direction of radial migration. Right panel: Sketch for the measurement of deviation angle. Black arrows indicate the direction of radial migration; green arrows show the migrating direction of traced neurons. Scale bar: 50 μm. (**f**) Percentage of each cell type in lower CP and IZ. *n *= 6 for each group. Data represent mean ± SEM. Student’s t- test; ***p < 0.001.

As Ezh2 is still expressed by postmitotic migrating neurons, to explore whether it is related to neuronal migration, loss-of-function studies were carried out by using Ezh2-specific short hairpin RNAs (Ezh2 shRNA1 and Ezh2 shRNA2), and a non-silencing shRNA with sequence not specific to any gene was used as a control. The knockdown efficiencies of the shRNAs were examined by transfecting them into cultured primary neurons. Western bolt analysis showed that both shRNAs could reduce endogenous Ezh2 protein level, and impaired its function manifested by a dramatic reduction of the chromatin status of H3K27me3 (Fig. 2a). As Ezh2 shRNA1 made stronger interference effect, it was used in most experiments. We conducted *in utero* electroporation at E14.5 using these shRNAs together with a GFP expression plasmid to identify transfected cells. It was confirmed that the intensity of Ezh2 immunofluorescence was obviously reduced in Ezh2 shRNA1-expressing neurons when analyzed at E17.5 (Fig. 2b).

Next, the distribution of GFP^+^ cells was analyzed at P0, at which time point the cortex could be divided into upper CP, lower CP, IZ and VZ/SVZ according to the nuclei density. Whereas the vast majority of neurons expressing control shRNA migrated into the upper CP, knockdown of Ezh2 led to accumulation of neurons in the IZ, and only a small portion of them reached the upper CP (Fig. 2c,d). Importantly, this migration impairment was greatly rescued by co-transfection of the Ezh2 shRNA1 resistant human EZH2 expression plasmid (hEZH2), as shown by a drastic increase of transfected neurons in the upper CP and an obvious reduction of them in the IZ (Fig. 2c,d), indicating that the phenomenon was indeed caused by Ezh2 deficiency. In addition, interference of Ezh2 by the less effective shRNA2 also induced an increased proportion of GFP^+^ cells in the IZ (see Supplementary Fig. S1a&b online). Moreover, immunofluorescence staining of Cux1, a neuronal marker for the upper CP, showed that its expression pattern was not altered by knocking down of Ezh2 (see Supplementary Fig. S1c online). Taken together, these results suggest that Ezh2 is required for cortical neuronal migration.

We further traced the morphology of neurons resided at the lower CP and IZ (Fig. 2e). Although a small number of neurons transfected with control shRNA were located in these regions, most of them were radially distributed and adopt the normal morphology of migrating cells characterized with a thick straight leading process pointing toward the pial surface of the cortex and a thin trailing process oriented to the VZ. While the Ezh2-knocked down neurons were more diversified in direction, manifested as with leading process deviated from the normal radial migrating direction. Especially for neurons resided in the IZ, many of them were tangentially distributed and assembled to each other. To make quantification of this phenomenon, deviation angle of leading process relative to the normal radial migration direction was measured (Fig. 2e). We defined cells with deviation angles less than 30**°** as radial cells, and cells with angles more than 150° as opposite cells, while others as oblique cells. It was illustrated that for Ezh2 knockdown neurons, most of them resided at lower CP belonged to opposite cells, while the proportion of oblique cells was substantially increased for those located at IZ (Fig. 2f). These results suggest that the radial migration direction of cortical neurons might be affected by Ezh2 deficiency and contribute to the defective migration.

### Ezh2 regulates neuronal orientation

**Figure 3:**
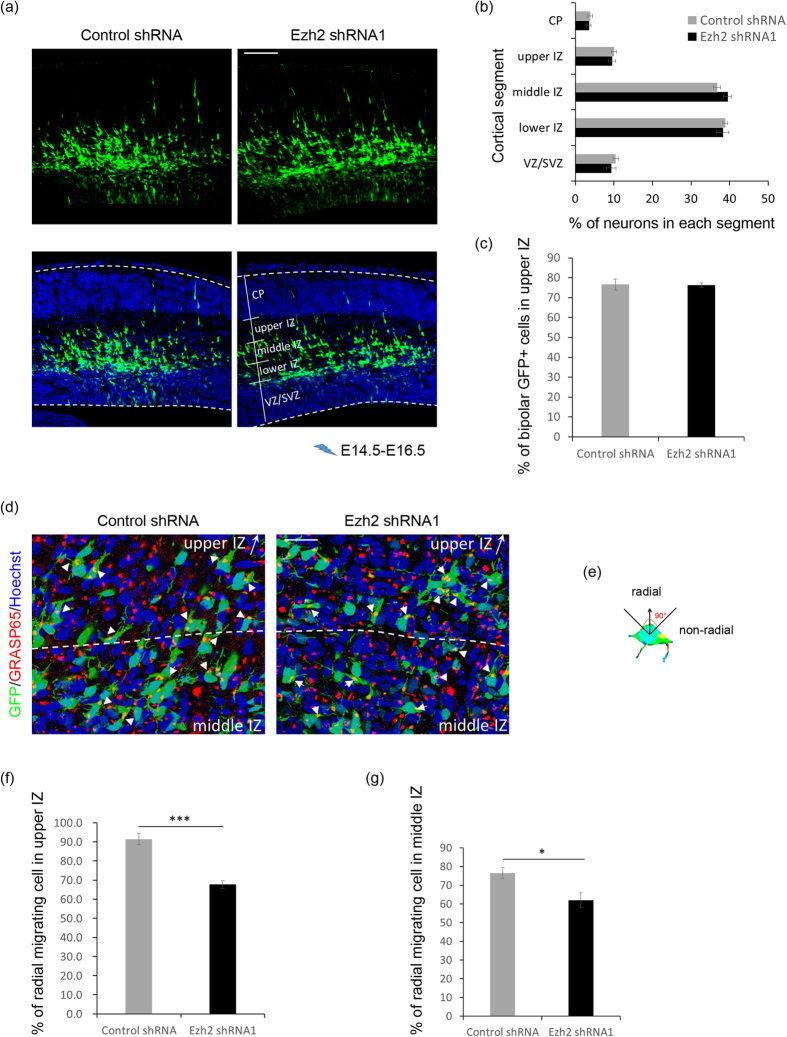
Ezh2 knockdown affects neuronal orientation. (**a**) Representative images showing the E14.5 mouse cortices electroporated with indicated plasmids and examined at E16.5. Scale bar: 100 μm. (**b**) Frequency distribution and quantification of GFP^+^ cells in different layers. *n *= 6 for each group. Data represent mean ± SEM. (**c**) Statistical analysis showing percentage of bipolar cells in upper IZ. *n *= 6 for each group. Data represent mean ± SEM. (**d**) Representative images showing the Golgi localization of electroporated neurons in the IZ (white arrows). Scale bar: 25 μm. (**e**) Scheme for the definition of radial and non-radial migrating cell by the location of Golgi apparatus (yellow). Black arrow points to the pial surface. (**f,g**) Statistical analysis showing percentage of radial migrating cells in each region. *n *= 6 for each group. Data represent mean ± SEM. Student’s *t* - test; *p < 0.05; ***p < 0.001.

Neurons generated at around E14.5 undergo series of transitions before they get to their final designations. After leaving the VZ, postmitotic neurons adopt a multipolar morphology and move in random direction in the SVZ and lower IZ^30^. Then axongenesis initiates in the middle IZ, followed by centrosomes and Golgi reorient toward the CP and cells begin to migrate radially^31^. When cells enter the upper IZ, they adopt a bipolar morphology and move by radial-glia guided locomotion to the CP^4^. To examine the migratory behavior affected by Ezh2-deficiency more closely, brain slices from E16.5 embryos electroporated at E14.5 were analyzed. As illustrated by Fig. 3a–c, both the distribution of GFP^+^ cells in each of the cortical regions and the ratio of bipolar cells in the lower region of upper IZ showed no difference between control and Ezh2 shRNA1-transfected neurons, which indicated that the multipolar to bipolar transition may not be affected by Ezh2 knockdown. Next we stained the Golgi protein GRASP65 to reflect the migratory direction of neurons in the upper and middle IZ, and defined radial migrating neuron as cell with Golgi apparatus located ahead of the nucleus and within 90° toward the pial surface (Fig. 3d). It was observed that the proportion of radial migrating bipolar neurons in the upper IZ and multipolar neurons in middle IZ were both significantly reduced in Ezh2 shRNA1 transfected brains (Fig. 3e–g), suggesting that Ezh2 deficiency affects neuronal orientation from early stage of radial migration.

### Ezh2 insufficiency induces ectopic expression of Reelin

**Figure 4:**
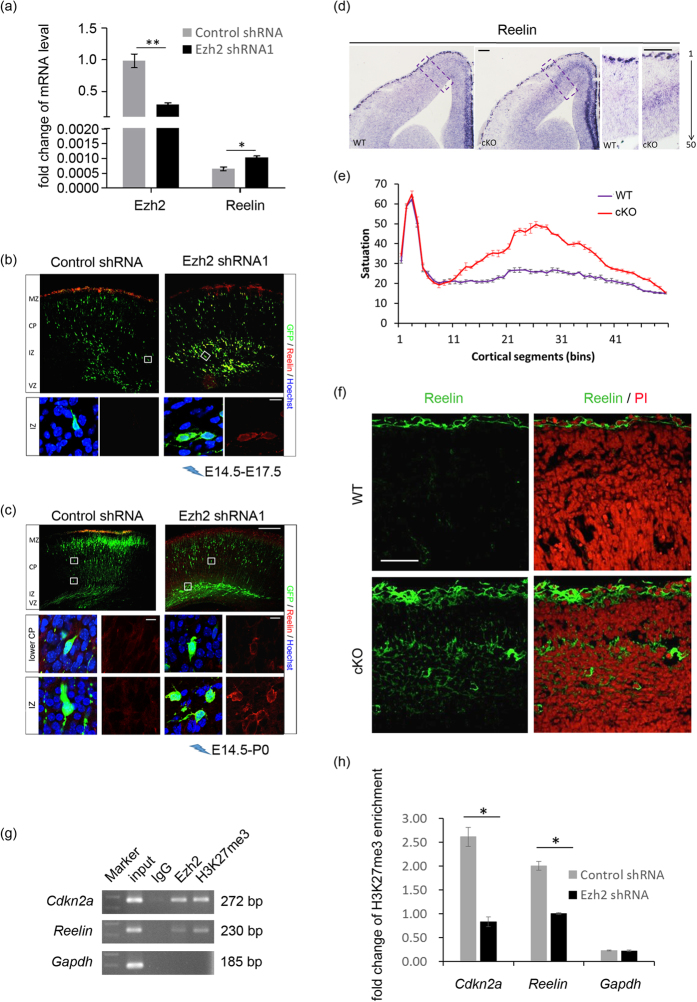
Ezh2 negatively regulates Reelin expression and binds to its promoter. (**a**) Real-time PCR confirms the decrease of Ezh2 mRNA level (left panel) and shows increase of Reelin mRNA level (right panel) in sorted Ezh2 shRNA1-transfected cells. Data were collected in triplicate, and are shown as mean ± SEM. Student’s *t*-test; *p < 0.05; **p < 0.01. (**b**) Immunofluorescence staining for Reelin on E17.5 sections showing ectopic Reelin expression in IZ. Scale bars: 200 μm (first row) and 10 μm (second row). (**c**) Immunofluorescence staining for Reelin on P0 sections showing ectopic Reelin expression in lower CP and IZ. Scale bars: 200 μm (first row) and 10 μm (second and third row). (**d**) *In situ* hybridization showing ectopic expression of Reelin in E14.5 Ezh2 cKO mouse brain section. Scale bars: 25 μm. (**e**) Quantification of Reelin signal saturation in fifty equal bins (CP 1 to VZ 50). *n *= 4 for each group. Data represent mean ± SEM. (**f**) Immunofluorescence staining showing ectopic expression of Reelin in E14.5 Ezh2 cKO mouse brain section. Scale bars: 50 μm. (**g**) PCR analysis of ChIP products from E16.5 cortex samples with indicated antibodies shows that both Ezh2 and H3K27me3 are enriched in *Reelin* promoter (cropped images, and full-length gel is presented in Supplementary Fig. S3b online). (**h**) Cultured E14.5 mouse cortical neurons were transfected with control shRNA or Ezh2 shRNA1 virus. Real-time PCR analysis of ChIP from above cells after 72 hours of transfection with H3K27me3 antibody shows reduced enrichment of that on *Reelin* promoter. Data were collected in triplicate, and are shown as mean ± SEM. Student’s *t*-test; *p < 0.05.

Previous study reported that in E12.5 Ezh2-null cortex, mRNA transcription level of Reelin in cortical progenitor cells was up-regulated by about 40% according to expression profile analysis^27^. Researches on Reelin functions also provided evidences that either insufficient or excess of this protein in embryo cortices both lead to severe developmental abnormalities^32^,^33^,^34^. Along with the migration deficiency and histological abnormality of Ezh2-knocked down neurons observed, we thus hypothesized that Ezh2 is required for the suppression of Reelin expression in radial migrating cortical neurons to maintain proper Reelin expression pattern. To test this, we first dissociated E16.5 embryo brains electroporated at E14.5 with control or Ezh2 shRNA1 plasmids. GFP^+^ cells were collected by FACS and the expression level of Reelin was measured. As indicated in Fig. 4a, in Ezh2-knocked down neurons, the mRNA level of Ezh2 was reduced by 70%, while there was a 60% increase in Reelin mRNA. The protein level of Reelin was also increased in these cells (see Supplementary Fig. S2a online). Immunofluorescence staining of Reelin on E17.5 brain sections that electroporated at E14.5 proved that besides normal expression in the marginal zone (MZ), where Cajal-Retzius neurons are located^35^, Reelin signal was also found in Ezh2-knocked down neurons in IZ (Fig. 4b). Furthermore, on P0 section, ectopic Reelin expression was still detectable in both lower CP and IZ-resided Ezh2-deficient cells (Fig. 4c).

Because *in utero* electroporation can only affect a small part of cortical neurons, to further determine the potential alteration pattern of Reelin expression induced by Ezh2 insufficiency, we performed *in situ* hybridization using probe specific for Reelin in wild type (WT) and Emx1-Cre mediated conditional Ezh2-knockout (cKO) mice. Both Ezh2 and H3K27me3 protein could be barely detected in E14.5 Ezh2 cKO cortices (see Supplementary Fig. S2b online), and the increase of Reelin mRNA level was also observed (see Supplementary Fig. S2c online). On E14.5 brain sections of WT mice, Reelin signals were limited to MZ while the they were also obviously detectable in the middle portion of the brain, presumably in the IZ, in Ezh2 cKO brains (Fig. 4d,e). Immunofluorescence of Reelin also proved this phenomenon (Fig. 4f). We therefore suggest that in the absence of Ezh2, Reelin is ectopically activated in the developing cortex, and this altered expression pattern may thereby lead to the mis-orientation of pre-migrating neurons and the migration defects.

As Ezh2 is the catalytic unit of PRC2 and mediates H3K27me3 at gene promoter regions, which results in transcription inactivation of targeted genes, we next performed chromatin immunoprecipitation (ChIP) to confirm the direct biding of Ezh2 and H3K27me3 on *Reelin* promoter. Because it has been verified in previous reports that Cdkn2a is not expressed in developing cortex whose promoter is enriched of H3K27me3 while Gapdh is constantly expressed and with promoter region occupied of gene activating marker^36^, we used *Cdkn2a* and *Gapdh* as positive and negative control, respectively, for H3K27me3 enrichment. ChIP of E16.5 WT cortices showed that by using anti-Ezh2 and anti-H3K27me3 antibody, promoter regions of *Reelin* and *Cdkn2a* but not *Gapdh* could be immunoprecipitated (Fig. 4g). Furthermore, in cultured neurons infected by Ezh2 shRNA1 expressing virus, H3K27me3 enrichment was significantly reduced on *Reelin* promoter (Fig. 4h). These results indicate that Ezh2 may regulate *Reelin* transcription by epigenetic mechanism that reduction of gene repression marker H3K27me3 on *Reelin* promoter by Ezh2 insufficiency leads to increased Reelin expression and ectopic distribution.

## Discussion

Precise neuronal migration is the basis for organizing correct brain architecture and essential for the establishment of specific neural circuits, which maintain functional connectivity of the brain. Radial migration of pyramidal is one important aspect, and is considered under subtle regulation of multiple intracellular and extracellular mechanisms including epigenetic regulation system^36^. In this study, we identified the role of transcription repressor Ezh2 in postmitotic migrating neurons that loss of Ezh2 function in these cells resulted in disturbed orientation, which further impaired the radial migration probably by alternating Reelin expression pattern in the cortex.

Previous studies have well described the role of Ezh2 in neurogenesis, but its function in otherwise neuronal development aspects still remain to be elucidated. Because brain structure abnormality, which might be the consequence of impaired neuronal migration, was reported in *EZH2* missense mutation-related Weaver syndrome patients^25^, we decided to explore the potential role of Ezh2 in neuronal migration *in vivo*. For the first step, the expression pattern of Ezh2 in cortical development was determined. In Pereira’s study, expression of Ezh2 was barely detected in E16 cortex^27^, while by using a monoclonal antibody we confirmed that Ezh2 is expressed throughout the period of cortical-genesis, and also showed that its expression was not only confined to progenitor cells but also in postmitotic migrating neurons.

In our study, Ezh2 knockdown by *in utero* electroporation at E14.5 resulted in apparent accumulation of neurons in the IZ at P0, and neurons resided in both lower CP and IZ showed varied or even inverted orientations, reminiscent of the phenotype exhibited in *reeler* mice in which *Reelin* was mutated^37^. Reelin is a matrix glycoprotein mainly expressed in Cajal-Retzius cells in the MZ, and has been reported to exert multiple functions during cortical radial neuronal migration^38^,^39^,^40^. The well-known function of Reelin in cortical neuronal migration is evidenced by the loss of “inside-out” pattern of neuronal migration^41^. Our results demonstrate that in Ezh2-deficient migrating neurons, expression of Reelin was abnormally initiated due to the reduction of H3K27me3 enrichment in the gene promoter region, and as a consequence the normal concentration gradient of Reelin cross the developing cortex might be disturbed. Thus it is likely that the alteration in neuronal orientation by knockdown of Ezh2 may be partially owing to the increased local concentration of Reelin. Previous study also reported that ectopic Reelin expression induced by directly *in utero* plasmid delivery or transplantation of Reelin-secreting cells into the developing cortex both lead to severe aggregation of migrating neurons^33^. While another research demonstrated that transgenetically generated ectopic Reelin expression in VZ in the presence of endogenous protein were not able to induce aggregate or apparent migration alteration^42^. The reason of the discrepancy among these studies might be explained by the variance of increased level of Reelin induced by different systems. Therefore, to maintain localized and homeostatic Reelin expression is of much importance for cortical morphogenesis. In normal conditions, Reelin secreted in the MZ binds to its receptors Vldlr and Apoer2 expressed by radial glia cells and migrating neurons, and activates downstream signaling pathways that make possible the correct neuronal migration pattern^32^,^43^,^44^. Further studies on how Reelin receptors may react in response to Ezh2 deficiency are still needed to make clear how Reelin signaling pathway may be affected by Ezh2-related chromatin remolding mechanism.

Besides cortical development, Ezh2 is reported to take part in other aspects in brain development through different pathways. For example, Ezh2 is involved in tangential migration of precerebellar pontine nuclei by represses Netrin1 in dorsal hindbrain and maintains spatially restricted Hox expression^45^, and Ezh2 regulates adult hippocampal neurogenesis through Pten-Akt-mTOR signaling pathway^46^. Together with our finding that Ezh2 regulates proper Reelin expression level in the developing cortex, Ezh2 plays multiple roles during nervous system development. More efforts may still be paid to evaluate Ezh2-implicated epigenetic regulation of neuronal development and its mutation-related neurological diseases.

In summary, our study provides new insight that polycomb chromatin modification component Ezh2 is involved in radial neuronal migration through epigenetically represses Reelin transcription in the migrating neurons to maintain appropriate Reelin expression pattern for proper orientation of these cells.

## Materials and Methods

### Plasmids

Plasmid expressing myc-tagged human EZH2 (hEZH2) was a kind gift from Prof. Mien-Chie Hung^47^ (The University of Texas MD Anderson Cancer Center, USA) and was subcloned to pCAGGS-EGFP vector with the cytomegalovirus enhancer element and chicken beta-actin promoter as well as enhanced green fluorescent protein. To generate short hairpin RNA (shRNA) vectors, oligonucleotides targeting mouse Ezh2 (NM_007971, shRNA1: 5′-GCAAATTCTCGGTGTCAAACA-3′ and shRNA2: 5′-GCTCCTCTAACCATGTTTACA-3′) were designed using BLOCK-iT^TM^ RNAi Designer (Invitrogen) and inserted into the pSUPER vector, and nonsilencing sequence 5′-GCAAATTCTCGGTGTCAAA-3′ was used as control shRNA. To create shRNA1-resistant EZH2 (hEZH2-R), synonymous mutations were performed for the third nucleotide of each codon in the target sequence.

### Animals

Pregnant mice used for *in utero* electroporation were purchased from Department of Laboratory Animal Science, Peking University Health Science Center. To generate Ezh2 conditional knockout mice, Ezh2^fl/fl^ mouse (B6;B6129-Ezh2 < tm1Yugo > /Hko, obtained from RIKEN Biomedical Center) was crossed with Emx1-Cre mouse (B6.129S2-Emx1tm1(cre)Krj/J, obtained from Jackson Laboratory). All animal experiments were approved by the institutional committee of Peking University and conducted according to the guidelines from the Ethics Committees of Peking University Health Science Center.

### *In utero* electroporation

Pregnant mice were deeply anesthetized with sodium pentobarbital and their uterine horns were exposed. Mixture of either shRNA plasmids (4 μg/μl) and pCAGGS-EGFP (4 μg/μl) or shRNA and pCAGGS-hEZH2R-EGFP (4 μg/μl) plasmids were prepared. Intrauterine embryos were carefully handled and 2 μl plasmid DAN with 0.01% Fast Green (Fluka) was injected into the lateral ventricles of the embryonic brain with a glass micropipette. Immediately after the DNA injection, electroporations of 5 times 50 ms, 36 V square pulses with 950 ms intervals generated by a BTX electroporator were delivered. The uterus was then replaced and the abdomen wall and skin were sutured. After surgical manipulation, mice were allowed to recover to conscious on a 37 °C incubator, and embryos were further developed for the time indicated.

### Primary neuron culture, transfection and lentivirus infection

E14.5 embryo cortices were isolated and digested with 0.25% trypsin (Hyclone) for 10 min at 37 °C, followed by dissociation in DMEM high glucose medium (Hyclone) containing 20% fetal bovine serum (Invitrogen). For electroporation, 3 × 10^6^ cells were nucleofected with 6 μg plasmids of control or Ezh2 shRNA as described by the manufacturer (Lonza). Neurons were plated on poly-D-lysine (Sigma Aldrich) coated 35 mm dishes and maintained at 37 °C and 5% CO2. Medium was replaced by Neurobasal medium (Invitrogen) containing 2% B27 supplement (Invitrogen) and 0.5 mM GlutaMAX-I (Invitrogen) 4 hours later. Cells for virus infection were plated on 60 mm dishes immediately after dissociation and treated as above, lentivirus containing control or Ezh2 shRNA1 sequence (GeneChem) was added at DIV1 with a multiplicity of infection of 1 and exchanged by fresh medium 24 hours later.

### Western Blotting

Cortices of indicated ages and primary cultured or sorted cells were lysed in ice-cold RIPA buffer containing protease inhibitors (Roche). Proteins were electrophoresed on 10% SDS-polyacrylamide gels and transferred to nitrocellulose membranes. Membranes were blocked with 5% non-fat milk in PBS-T (0.05% Tween-20) buffer for 60 min, and incubated with primary antibody at 4 °C overnight. After washing 3 times with PBS-T, membranes were incubated for 1 h at room temperature with secondary antibodies. Immunoreactive bands were then detected and analyzed by Odyssey Infrared Imager (LICOR Bioscience).

### Immunostaining

Brains were fixed in 4% paraformaldehyde in PBS overnight, cryoprotected in 25% sucrose, embedded in O.C.T. compound (tissue-Tek), and then frozen for sectioning with a Leica 3050S cryostat. Immunostaining was performed on 25 μm coronal sections. After citrate antigen retrieval, sections were blocked and permeabilized in 4% bovine albumin in 0.1M PBS with 0.3% Triton X-100, incubated in primary antibodies at 4 °C overnight and appropriate fluorescent secondary antibodies for 2 h at room temperature. Finally, sections were counterstained with Hoechst 33342 (Sigma-Aldrich) or propidium iodide (PI, Invitrogen) and coverslips were applied. Images were acquired by either FV-1200 (Olympus) or TCS SP5 (Leica) confocal laser scanning microscope.

### EdU labeling

For *in vivo* EdU labeling, pregnant mice were intraperitoneally injected with a 50 μg/g body weight dose of EdU (Invitrogen). Two hours or 48 hours after administration, pregnant mice were anesthetized and embryos were taken out for fixation. Frozen sections for the visualization of EdU labeled cells were reacted by the Click-iT kit (Invitrogen) with Alexa Fluor 647 azide for 30 min.

### FACS, RNA isolation and RT-qPCR

Cerebral hemispheres of E16.5 embryos electroporated with control or Ezh2 shRNA1 at E14.5 were dissociated under a fluorescence-dissecting microscope to precisely visualize the labeled regions. Cells were digested, proceeded to FACS, and GFP^+^ cells were purified. Total RNA was isolated using the RNeasy Plus Mini Kit for RNA isolation (Qiagen) according to the manufacturer’s instructions and cDNA was synthesized with reverse transcriptase (Fermentas). The quantitative PCR reactions were performed using SYBR Green master mix (Takara) on ABI PRISM 7300 System (Applied Biosystems). The amplification conditions were 2 min initial denaturation at 95 °C, followed by 40 cycles of each 5 s at 95 °C and 1 min at 60 °C, and samples were run in triplicate. The relative gene expression between samples was normalized with β-actin. Primers used were: Reelin: 5′-TTACTCGCACCTTGCTGAAAT-3′ (forward), 5′-CAGTTGCTGGTAGGAGTCAAAG-3′ (reverse); Ezh2: 5′-AACAGTAGCAGACCCAGCACCC-3′ (forward), 5′-CAGCACCACTCCACTCCACATT-3′ (reverse); β-actin: 5′-GCAAGCAGGAGTACGATGAG-3′ (forward), 5′-CCATGCCAATGTTGTCTCTT-3′ (reverse).

### ChIP assay

ChIP was performed using EZ-ChIP™ Kit (Millipore) according to the manufacturer’s instructions. Cross-linked sonicated chromatin was equally divided and immunoprecipitated with indicated antibodies. Quantitative PCR was carried out using the ABI PRISM 7300 System (Applied Biosystems), and the amounts of immunoprecipitated DNA were normalized to the input. Gene promoter regions were identified by promoter clones ( http://www.genecopoeia.com). Primers for the promoter regions were *Reelin*: 5′-AAACGTGCTTCTGGATGGTT-3′ (forward), 5′-CTTCGCCGGACTCTGTATTT-3′ (reverse); *Cdkn2a*: 5′-GGGGCTGTCCGATCCTTTAG-3′ (forward), 5′-CCCAGTCGTGTGACCAGT-3′ (reverse); *Gapdh*: 5′-CACCATCCGGGTTCCTATAAATAC-3′ (forward), 5′-CAGCATCCCTAGACCCGTACA-3′ (reverse).

### Antibodies

Primary antibodies used were rabbit anti-Ezh2 (Cell Signaling Technology), rabbit anti-H3K27me3 (Cell Signaling Technology), rabbit anti-α Tubulin (Abcam), rabbit anti-H3 (Cell Signaling Technology), mouse anti-Reelin (Millipore), rabbit anti-GRASP65 (Abcam), rabbit anti-Cux1 (Santa Cruze), rabbit normal IgG (Applygen). Second antibodies included anti-rabbit IgG IRDye 680 and anti-mouse IgG IRDye 800 (LICOR Bioscience) for western blot and Alexa 488 anti-rabbit IgG, alexa 555 anti-rabbit IgG (Invitrogen) for immunostaining.

### *In Situ* Hybridization

*In situ* hybridization was performed with digoxigenin-labeled (Roche) riboprobes by standard protocol^48^. Sequences for antisense probe targeting mouse Reelin mRNA (Allen Brain Atlas) were amplified from cDNA library and cloned to pGEM-T vector (Promega). Images were acquired with an Olympus IX73 microscope and analyzed with Metamorph Software.

### Statistics

Data were show as mean ± SEM and tested for statistical significance by two-tailed Student’s *t*-test or one-way ANOVA followed by Tukey’s multiple comparison tests. Values of *p *< 0.05 were considered statistically significant, and asterisks were marked as *p < 0.05, **p < 0.01, ***p < 0.001.

## Additional Information

**How to cite this article**: Zhao, L. *et al.* Ezh2 is involved in radial neuronal migration through regulating Reelin expression in cerebral cortex. *Sci. Rep.* **5**, 15484; doi: 10.1038/srep15484 (2015).

## Supplementary Material

Supplementary Information
