# Supplementary material for: Ezh2 is involved in radial neuronal migration through regulating Reelin expression in cerebral cortex
Source: Sci Rep. 2015 Oct 26;5:15484. doi: 10.1038/srep15484 (PMC4620455; doi:10.1038/srep15484)
Supplement: Supplementary Information [file srep15484-s1.pdf]

Supplementary Information

Ezh2 is involved in radial neuronal migration through regulating Reelin expression in cerebral cortex

Linnan Zhao[1,2], Jun Li[1,2], Yuanlin Ma[1,2], Jiutao Wang[3], Wen Pan[4], Kai Gao[1,2], Zhengrong Zhang[1,2], Tianlan Lu[1,2], Weihua Yue[1,2], Shanting Zhao[3], Lifang Wang[1,2,*] & Dai Zhang[1,2,5,6,*]

[1] Peking University Sixth Hospital/Institute of Mental Health, Beijing 100191, China; [2] National Clinical Research Center for Mental Disorders / Key Laboratory for Mental Health, Ministry of Health (Peking University), Beijing 100191, China; [3] College of Veterinary Medicine, Northwest A&F University, Yangling 712100, China; [4] National Laboratory of Biomacromolecules, Institute of Biophysics, Chinese Academy of Sciences, Beijing 100101, China; [5] Peking-Tsinghua Center for Life Sciences and PKU-IDG/McGovern Institute for Brain Research, Peking University, Beijing 100871, China; [6] Shenzhen Key Laboratory for Neuronal Structural Biology, Shenzhen Peking University-Hong Kong University of Science and Technology Medical Center, Shenzhen 518035, China.

[*] Corresponding author: Prof. Dai Zhang, M.D., E-mail: daizhang@bjmu.edu.cn or Prof. Lifang Wang, PhD., E-mail: lifangwang@bjmu.edu.cn. Institute of Mental Health, Peking University, 51 Hua Yuan Bei Road, Beijing 100191, P.R. China.

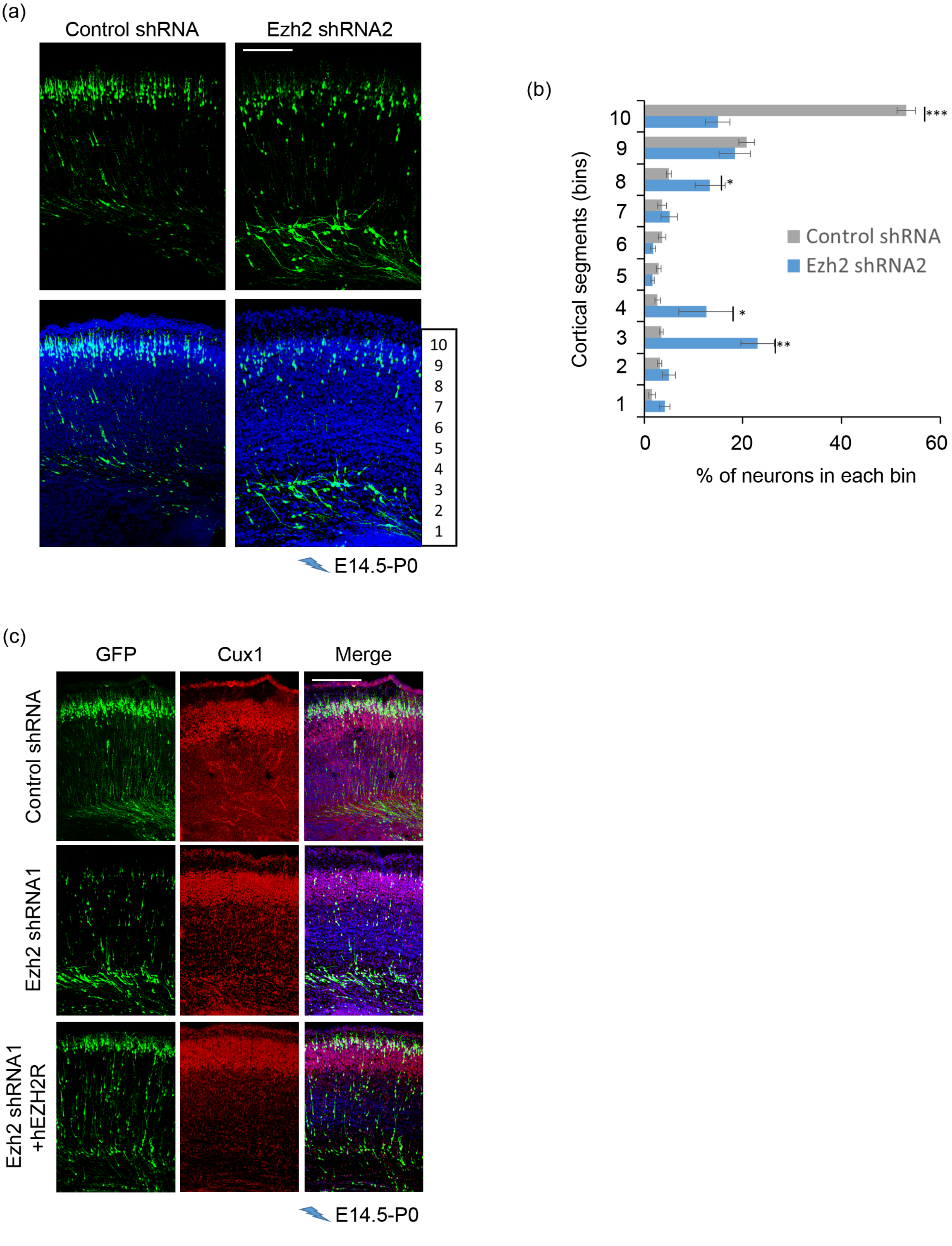

Supplementary Fig. S1. Knockdown of Ezh2 impairs neuronal migration.
(a) Representative images showing the E14.5 mouse cortices electroporated with indicated plasmids and examined at P0. Ezh2-knock down by shRNA2 leads to a reduction in the number of neurons in the CP, while an accumulation of neurons in the IZ.
(b) Frequency distribution and quantification of GFP$^+$ cells in ten equal bins (VZ 1 to CP 10). n=6 for each. Data represent mean ± SEM. Stutent's t-test; *p < 0.05; **p<0.01; ***p<0.001. Scale bar: 200 μm.
(c) Representative images showing the immunofluorescence staining for Cux1. Ezh2-knock down by shRNA1 does not affect the neuronal property. Scale bar: 200 μm.

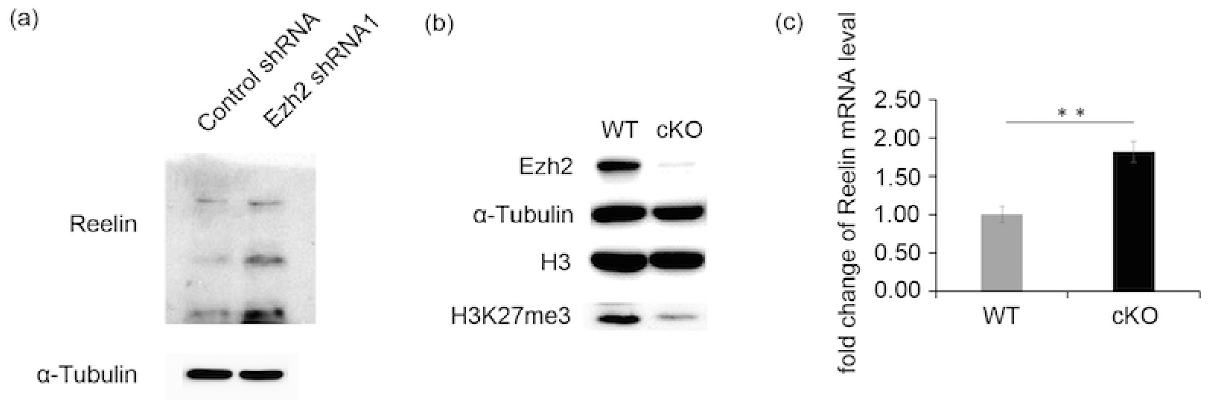

Supplementary Fig. S2. Ezh2 negatively regulates Reelin expression.
(a) Representative images of western blotting showing increased Reelin (multi bands between 180 - 400 kDa after long time exposure) expression in sorted Ezh2 shRNA1-transfected cells.
(b) Representative images of western blotting showing decreased Ezh2 and H3K27me3 level in Ezh2 cKO cortices.
(c) Real-time PCR analysis shows the increasement of Reelin mRNA level in Ezh2 cKO cortices. Data we re collected in triplicate, and are shown as mean ± SEM. Student's *t*-test; $^{**}p<0.01$.

(a)

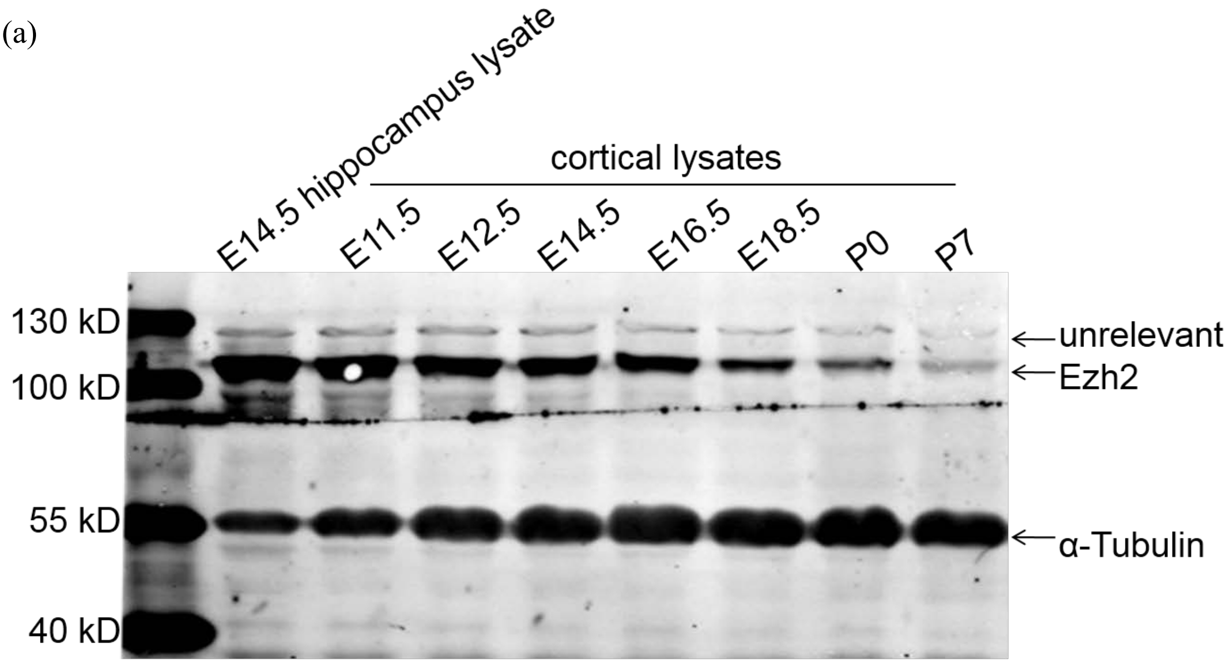

(b)

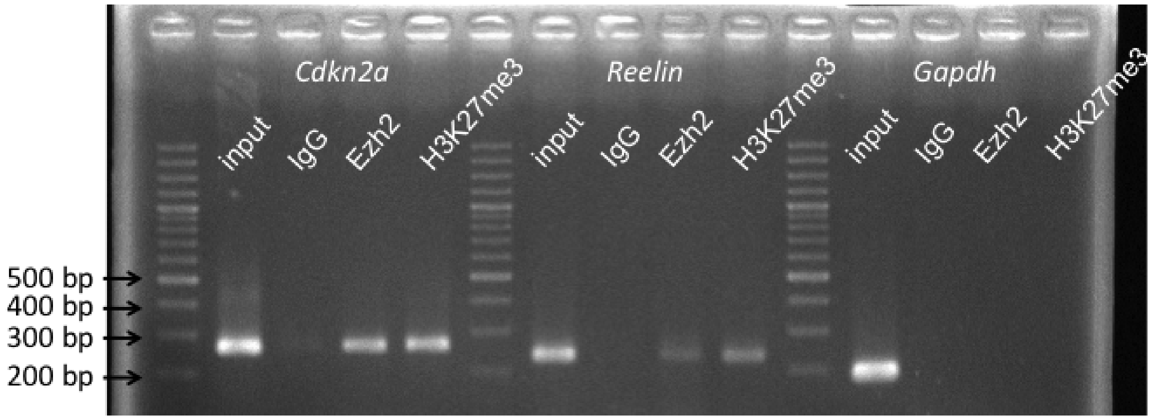

Supplementary Fig. S3. Full length images.
(a) Full length image for Figure 1a showing the immunoblotting for Ezh2 in mouse cortex lysates at the indicated ages.
(b) Full length image for Figure 4g showing the PCR analysis of ChIP products from E16.5 cortex samples with indicated antibodies.
